# Photoinduced Synthesis of New Diisochromenochromen-4-ones and Their Antimicrobial Activities

**DOI:** 10.1100/2012/954934

**Published:** 2012-05-01

**Authors:** Mohamad Yusuf, Indu Solanki, Payal Jain

**Affiliations:** Department of Chemistry, Punjabi University, Punjab, Patiala147002, India

## Abstract

The diisochromenochromen-4-one **3a**-**3b**, **4a**-**4c**, **5a**-**6a** **& 7** have been prepared from the photocyclization reaction of bischromen-4-one **2a**-**2e**. The later compounds are obtained from the O-alkylation of the suitable 3-hydroxy-2-aryl-4*H*-chromen-4-one **1a**-**1e** with 4,4′-bischloromethyl-diphenyl in dry acetone, anhydrous K_2_CO_3_, and PTC (Bu_4_N^+^I^−^) under refluxing conditions. The structures of compounds **2a**-**2e**, **3a**-**3b**, **4a**-**4c**, **5a**-**6a** **& 7** have been characterized from the rigorous analysis of their IR, ^1^H-NMR, ^13^C-NMR, ESI-Mass, and elemental analysis. The antibacterial and antifungal activities of the synthesized products were also evaluated against the *Klebsiella pneumoniae, Pseudomonas aeruginosa, Escherichia coli, Staphylococcus aureus, Bacillus subtilis*, and *Aspergillus janus* and *Penicillium glabrum*, respectively. Some of the tested compounds showed significant activity against the above-said microorganisms.

## 1. Introduction

Syntheses of six membered heterocyclic compounds have been the subject of major interest for researchers due to their significant biological activities [1–12]. The photochemical reaction of C=O compounds leads to the formation of many exotic carbocyclic and heterocyclic compounds, and these reactions are initiated through the intramolecular H-abstraction by the photoexcited carbonyl group from the *γ* and *δ* position to give 1, 4 and 1, 5-biradicals which finally collapse to the generation of many unique heterocyclic products [13–20]. 2-Aryl-3-alkoxy-chromen-4-one is such substrates which can undergo *γ* H-abstraction to provide pyran derivatives from the cyclisation reaction of 1, 4-biradical and 2-aryl ring [21–23]. The bischromen-4-one is the bichromophoric molecules which are formed by joining two chromen-4-one moieties together through the carbon chain of varying length and structure, and photochemical reaction of these compounds may produce some interesting heterocyclic compounds. Generally, the heterocyclic compounds are obtained via multistage reaction in the presence of specific reagents and reaction conditions. We are aiming hereby to synthesize bispyran derivatives under the influence of light. This aspect has prompted us to investigate the synthesis of diisochromeno-chromen-4-ones from the photochemical reaction of bischromen-4-one **2a**-**2e **built around the diphenyl spacer moiety. The major interest behind this study was to investigate the simple method and antimicrobial evaluations of new diisochromeno-chromen-4-ones. 

## 2. Results and Discussion

**Scheme 1:**
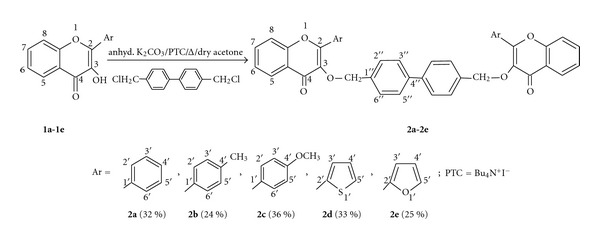


The compound **2a**-**2e **required for this study was obtained from the O-alkylation of the suitable 3-hydroxy-chromen-4-one [24–26] with 4,4′-bischloromethyldiphenyl in the presence of anhydrous K_2_CO_3_ and Bu_4_N^+^I^−^ (PTC) in dry acetone (Scheme 1). The reactions carried out in the absence of PTC provided very poor yield of the bischromen-4-ones and also involved very long reaction times. The monoalkoxy chromen-4-one formed in these reactions was removed by using column chromatography (60–120 mesh). The structures of **2a-2e** were determined by means of their IR, ^1^H-NMR, and ESI-MS spectral data (see the appendix).

The IR spectra of **2a-2e** exhibited strong absorptions in the region of 1640–1650 cm^−1^ which indicated the presence of conjugated C=O group. The major feature of their ^1^H-NMR spectra was the appearance of sharp singlet at *δ* 5.11–5.35 which may be assigned to 3-OCH_2_ group. The downfield appearance of this proton could be ascribed to their benzylic nature and placement near an electronegative oxygen atom. ^13^C-NMR of these compounds revealed the most significant downfield signal at *δ* 176.13–175.01 (C=O group), another signal at *δ* 142.12–139.08 due to C-3 due of its direct linkage to oxygen atom, and remaining aromatic carbon atoms were resonating at *δ* 161.47–117.97. The most upfield resonance was found placed at *δ* 72.07–69.02 due to OCH_2_ group.

**Scheme 2:**
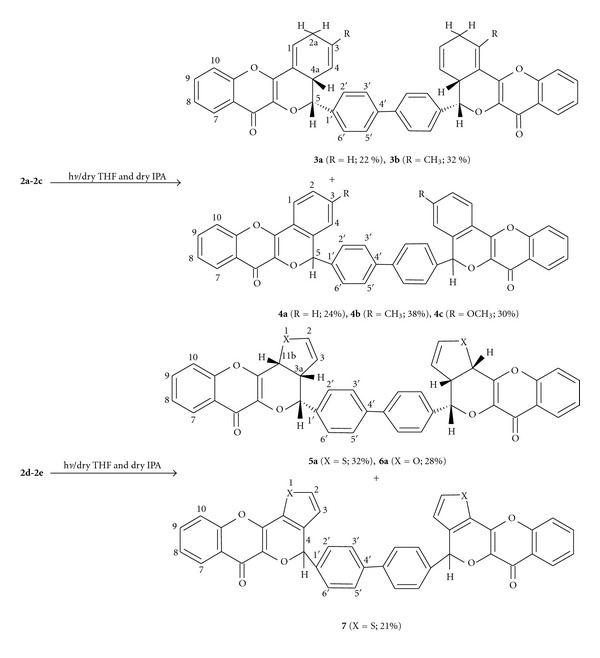


The photochemical reaction of bischromen-4-ones **2a-2e** was carried out under inert atmosphere in dry MeOH and THF (1 : 1) with Pyrex-filtered light from a 125 W Hg arc lamp. The progress of the photoreactions was monitored by TLC, and after about 8–10 hrs most of the starting compound was transformed to new products (Scheme 2). The column chromatographic separation of the reaction mixtures yielded **3a-3b**, **4a-4c**, **5a**, **6a**, and **7** in moderate yields. The structure of these products became evident from the comparison of their IR, ^1^H-NMR, and ^13^C-NMR spectra with those of starting compound **2a-2e** (see the appendix).

The appearance of one IR absorption band in the carbonyl group region of 1630–1650 cm^−1^ indicates that these products are obtained involving the both side cyclization on the bischromen-4-one. The ^1^H-NMR spectra of **3a** and **3b** were quite informative which gave broad singlet at *δ* 6.81–6.78 (H-1), dd at *δ* 5.85–5.80 (H-3), and another dd at *δ* 5.30–5.25 (H-4). The noticeable signals were centered at *δ* 5.01–5.05 (H-5) and 3.40–3.45 (H-4a) having coupling value of 11.0 Hz which describes their *cis* disposition at C-4a and C-5. The four-proton multiplet found at *δ* 2.95–2.92 may be assigned to H-2a. ^13^C-NMR spectra of these compounds were also very helpful which showed resonances at *δ* 178.20–177.82 due to carbonyl group, at *δ* 39.00–38.80 and *δ* 32.00–31.05 due to C-4a and C-2, respectively. Another signal at *δ* 126.53 could be assigned to C-1 which was absent in their starting compounds.

The ^1^H-NMR spectrum of **4a**-**4c** was very simple which produced most of the resonances in aromatic region at *δ* 8.30–6.95, and a significant singlet at *δ* 5.85–5.78 (2H) may be allotted to H-5. ^13^C-NMR of these compounds showed the suitable signals due to aromatic carbon in the region of *δ* 156.68–114.58, and the signals at *δ* 72.93–71.74 could be resulted by C-5.

Similarly the major features of ^1^H-NMR spectra of **5a**-**6a** were the signals due to H-3a, H-11b, &H-4 which clearly resonating at *δ* 3.70–3.67 (2H, ddd), 5.11–5.06 (2H, d), and 5.48-5.45 (2H, d), respectively. The coupling value of *J*_3a,11b_ = 8.1 Hz and *J*_3a,4_ = 10.0 Hz describes the *cis* relationship both between H-3a & H-11b and H-3a & H-4. In the ^13^C-NMR spectra, suitable resonances were found to be placed at *δ* 178.20–177.62, 83.30–82.10, 47.20–47.12 and 34.50–33.48 which may be very well represented by C=O, C-4, C-3a, and C-11b, respectively. The various spectral data also fully confirmed the structural features of the compound **7**.

**Scheme 3:**
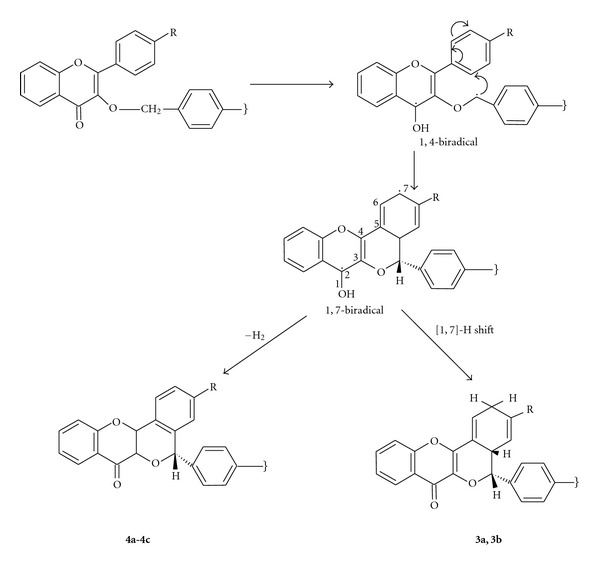
Mechanism of the photocyclizations of bischromon-4-ones **2a-2c**.

Mechanistically, the phototransformation of bischromen-4-ones **2a-2c** may be occurring through the H-abstraction by the photoexcited C=O group from the 3-benzyloxy group to give 1,4-biradical which undergoes cyclization with the 2-aryl ring to provide 1,7-biradical. The later may suffer [1,7]-H shift to give **3a-3b** while oxidation of the biradical produces **4a-4c **(Scheme 3). Similarly, the formation of the products **5a-6a **and** 7** from the photocyclization of bischromon-4-ones **2c-2d** may also be described.

## 3. Antimicrobial Activity

The antimicrobial activity of synthesized compounds was screened in vitro against selected pathogens which include *Staphylococcus aureus *(MTCC 96),* Bacillus subtilis *(MTCC 441)*, Escherichia coli *(MTCC 443),* Pseudomonas aeruginosa* (MTCC 424),* and Klebsiella pneumoniae *(MTCC 3384), and fungus strains were* Aspergillus janus* (2751) and* Penicillium glabrum *(4951). All the compounds were also screened for MIC by using serial tube dilution method [27] at concentration 3.12, 6.25, 12.5, 25, 50, and 100 *μ*g/mL against the above-said microorganisms, and observed minimum inhibitory concentration (MIC-*μ*g/mL) values are given in Tables 1 and 2. Compounds **2a**, **2d**, and **2e** showed significant activity against* Escherichia coli* and* Klebsiella pneumoniae* whereas **2b **and **2c** showed significant activity against *Pseudomonas aeruginosa, Staphylococcus aureus*, and* Pencilluim glabrum*. The photoproducts **3a**, **3b**, **4a**, **5a**, and **6a** also exhibited significant activity against *Escherichia coli*, *Klebsiella pneumoniae*, *Pseudomonas aeruginosa*, *Staphylococcus aureus*, and* Pencilluim glabrum*, respectively. It is evident from the above study that 2-phenyl/thienyl/furanyl-bischromen-4-ones seem to be better antimicrobial agents than 2-tolyl/anisyl-bischromen-4-one derivatives. The importance of this work lies in the possibility that newly prepared compounds (**2a**, **2b**, **2c**, **2d**, **3a**, **3b**, **4a**, **5a**, and **6a**) might be more efficacious derivatives against the above-said bacterial and fungal strains.

## 4. Conclusion

This study provides the photochemical method for the preparation of new diisochromenochromen-4-one linked through the diphenyl moiety. The products have been obtained without using any specific and toxic reagent. The antimicrobial analysis of the prepared compounds has also been carried out and the importance of this work lies in the possibility that newly synthesized compounds (**2a**, **2b**, **2c**, **2d**, **3a**, **3b**, **4a**, **5a**, and **6a**) might be more efficacious derivatives against the tested bacterial and fungal strains. The investigations regarding the more biological studies of these bischromen-4-ones could be helpful in designing the potent antimicrobial agents.

## Figures and Tables

**Table 1:** In vitro antimicrobial MIC (*μ*g/mL) of compounds **2a-2b**.

Compound No.	Gram-negative bacteria			Gram-positive bacteria		Fungi	
	*Escherichia coli*	*Klebsiella pneumonia*	*Pseudomonas aeruginosa*	*Staphylococcus aureus*	*Bacillus subtilis*	*Aspergillus janus*	*Penicillium glabrum*
**2a**	12.5	12.5	50	25	25	50	25
**2b**	50	25	12.5	25	12.5	25	50
**2c**	25	25	12.5	12.5	25	25	12.5
**2d**	12.5	12.5	25	25	50	12.5	12.5
**2e**	12.5	12.5	12.5	25	25	25	12.5
Amoxicillin	3.12	3.12	3.12	3.12	3.12		
Fluconozole	—	—	—	—	—	3.12	3.12

**Table 2:** In vitro antimicrobial MIC (*μ*g/mL) of photoinduced compounds **3a-3b, 4a-4c, 5a-6a & 7**.

Compound No.	Gram-negative bacteria			Gram-positive bacteria		Fungi	
	*Eschericha coli*	*Klebsiella pneumonia*	*Pseudomonas aeruginosa*	*Staphylococcus aureus*	*Bacillius subtilis*	*Aspergillus janus*	*Penicillium glabrum*
**3a**	12.5	12.5	50	12.5	50	25	12.5
**3b**	25	25	25	25	12.5	12.5	12.5
**4a**	12.5	50	12.5	12.5	12.5	12.5	12.5
**4b**	50	25	25	50	25	50	25
**4c**	25	12.5	25	25	25	12.5	50
**5a**	12.5	25	12.5	25	25	25	50
**6a**	50	12.5	25	12.5	12.5	12.5	12.5
**7**	12.5	25	50	25	25	25	12.5
Amoxicillin	3.12	3.12	3.12	3.12	3.12		
Fluconozole	—	—	—	—	—	3.12	3.12

## Appendix

### A. Experimental

#### A.1. Synthesis of 3,3′-(Biphenyl-4,4′-Diylbis(Methylene))Bis(Oxy)Bis(2-Phenyl-4*H*-Chromen-4-One) **2a**

The bischromenone **2a** was synthesized by reacting compound **1a** (2.00 g, 0.0089 mole) with 4,4′-bischloromethyl-diphenyl (1.12 g, 0.0044 mole) in presence of anhyd. K_2_CO_3_ and Bu_4_N^+^I^−^ as PTC in dry acetone. Progress of reaction was monitored by TLC. After the completion of reaction, reaction mixture was turned into colorless mass which was poured into iced HCl to obtain a crude product which was recrystallized with CHCl_3_ : MeOH to yield pure bischromone **2a**.

Colorless solid; yield 32%; m.p.: 180–182°C; IR (KBr) cm^−1^: 1640 (C=O), 1616 (C=C); ^1^H-NMR (400 MHz, DMSO-d_6_): *δ* 8.32 (2H, dd, *J*_p,o_ = 1.6, 8.0 Hz, H-5), 7.99 (4H, dd, *J*_o_ = 1.5, 5.8 Hz, H-2′, 6′), 7.69 (2H, m, H-7), 7.53 (2H, d, *J*_o_ = 8.3 Hz, H-6), 7.46 (12H, m, H-8, 4′, 2′′, 3′′, 5′′, 6′′), 7.34 (4H, d, *J*_o_ = 8.3 Hz, H-3′, 5′), 5.16 (4H, s, OCH_2_); ^13^C-NMR (DMSO-d_6_): *δ* 176.04 (C-4), 155.23 (C-2), 153.02 (C-8a), 139.08 (C-3), 139.00 (C-4′′), 136.04 (C-1′′), 135.82 (C-7), 130.30 (C-1′), 128.51 (C-3′, 5′), 128.10 (C-3′′, 5′′), 127.9 (C-2′, 4′, 6′), 127.6 (C-2′′, 6′′), 125.71 (C-4a), 124.21 (C-5), 123.42 (C-6), 113.64 (C-8), 72.02 (OCH_2_); MS (ESI): m/z (M)^+^ = 655; Anal. Calc. For C_44_H_30_O_6_: C, 80.73; H, 4.58; found C, 80.69; H, 4.57%.

#### A.2. Synthesis of 3,3′-(Biphenyl-4,4′-Diylbis(Methylene))Bis(Oxy)Bis(2-p-Tolyl-4*H*-Chromen-4-One) **2b**

The bischromenone **2b **was obtained from the reaction of compound **1b** (2.00 g, 0.0079 mol) with 4,4′-bischloromethyl-diphenyl (0.99 g, 0.0039 mole) under similar condition as described above for **2a**.

Colorless solid; yield 24%; m.p.: 208–210°C; IR (KBr) cm^−1^: 1642 (C=O), 1611 (C=C); ^1^H-NMR (400 MHz, DMSO-d_6_): *δ* 8.30 (2H, dd, *J*_p.m_ = 1.4, 7.9 Hz, H-5), 7.93 (4H, d, *J*_o_ = 8.3 Hz, H-2′,6′), 7.66 Hz (2H, ddd,  *J*_p.m.o._ = 0.9, 1.6, 8.6 Hz, H-7), 7.51 (2H, d, *J*_o_ = 8.4 Hz, H-6), 7.45 (2H, d, *J*_o_ = 8.2 Hz, H-8), 7.40 (8H, m, H-2′′, 3′′, 5′′, 6′′), 7.27 (4H, d, *J*_o_ = 8.2 Hz, H-3′, 5′), 5.12 (4H, s, 3-OCH_2_), 2.44 (3H, s, 4′-CH_3_); ^13^C-NMR (DMSO-d_6_): *δ* 175.16 (C-4), 156.78 (C-8a), 155.32 (C-2), 141.13 (C-3), 140.61 (C-4′), 139.62 (C-4′′), 135.81 (C-1′′), 133.40 (C-7), 129.32 (C-3′, 5′), 129.08 (C-1′), 128.80 (C-3′′, 5′′), 128.18 (C-2′′, 6′′), 126.92 (C-2′, 6′), 125.81 (C-5), 124.69 (C-6), 124.24 (C-4a), 118.06 (C-8), 73.71 (OCH_2_), 21.61 (4′-CH_3_); MS (ESI): m/z (M)^+^ = 683; Anal. Calc. For C_46_H_34_O_6_: C, 80.93; H, 4.98%; found C, 80.86; H, 4.97%.

#### A.3. Synthesis of 3,3′-(Biphenyl-4,4′-Diylbis(Methylene))Bis(Oxy)Bis(2-(4-Methoxyphenyl)-4H-Chroman-4-One) **2c**

The bischromenone **2c **was synthesized from the reaction of compound **1c** (2.00 g, 0.0074 mole) with 4,4′-bischloromethyl-diphenyl (0.93 g, 0.0037 mole) under similar condition as described above for **2a**.

Colorless solid; yield 36%; m.p.: 194–196°C; IR(KBr) cm^−1^: 1638 (C=O), 1603 (C=C);^ 1^H-NMR (400 MHz, DMSO-d_6_): *δ* 8.20 (2H, dd, *J*_p.o_ = 1.4, 7.9 Hz, H-5), 8.04 (4H, dd, *J*_p,o_ = 1.8, 7.0 Hz, H-2′,6′), 7.74 (2H, ddd, *J*_p.m.o_ = 1.0, 2.0, 7.6 Hz, H-7), 7.60 (2H, d, *J*_o_ = 8.3 Hz, H-6), 7.51 (4H, d, *J*_o_ = 8.3 Hz, H-3′′, 5′′), 7.43 (6H, m, H-8, 2′′, 6′′), 7.00 (4H, dd, *J*_p,o_ = 1.8, 7.0 Hz, H-3′, 5′), 5.11 (4H, s, 3-OCH_2_), 3.88 (6H, s, 4′-OCH_3_);^ 13^C-NMR (DMSO-d_6_): *δ* 175.07 (C-4), 161.47 (C-8a), 156.61 (C-4′), 155.23 (C-2), 140.59 (C-1′′), 139.18 (C-3), 135.82 (C-7), 133.32 (C-5), 130.62 (C-3′′, 5′′), 129.36 (C-2′′, 6′′), 126.90 (C-2′, 6′), 125.79 (C-4a), 124.66 (C-6), 124.21 (C-4′′), 123.37 (C-1′), 117.97 (C-3′, 5′), 113.75 (C-8), 73.67 (OCH_2_), 55.45 (4′-OCH_3_); MS (ESI): m/z (M)^+^ = 715; Anal. Calc. For C_46_H_34_O_4_: C, 77.31%; H, 4.76%; found C, 77.54; H, 4.77%.

#### A.4. Synthesis of 3,3′-(Biphenyl-4,4′-Diylbis(Methylene))Bis(Oxy)Bis(2-(Thiophen-2-yl)-4*H*-Chromen-4-One **2d**

The bischromenone **2d **was synthesized from the reaction of compound **1d** (2.00 g, 0.0087 mole) with 4,4′-bischloromethyl-diphenyl (1.10 g, 0.0043 mole) under similar condition as described above for **2a**.

Colorless solid; yield 33%; m.p.: 225–227°C; IR (KBr) cm^−1^: 1641 (C=O), 1603 (C=C); ^1^H-NMR (400 MHz, DMSO-d_6_): *δ* 8.22 (2H, dd, *J*_m,o_ = 1.7, 8.0 Hz, H-5), 7.96 (2H, d, *J*_3′,4′_ = 3.7 Hz, H-3′), 7.72 (2H, ddd, *J*_p,m,o_ = 1.2, 2.4, 7.8 Hz, H-7), 7.65 (2H, d, *J*_5′,4′_ = 4.8 Hz, H-5′), 7.62 (4H, d, *J*_o_ = 8.0 Hz, H-3′′, 5′′), 7.54 (6H, m, H-8, 2′′, 6′′), 7.48 (2H, d, *J*_o_ = 8.0 Hz, H-6), 7.20 (2H, dd, *J* = 3.7, 4.8 Hz, H-4′), 5.35 (4H, s, 3-OCH_2_), ^13^C-NMR (DMSO-d_6_): *δ* 176.13 (C-4), 157.21 (C-8a), 142.12 (C-3), 140.14 (C-1′′), 136.93 (C-2), 135.52 (C-4′′), 135.3 (C-7), 130.61 (C-5), 130.53 (C-5′), 129.08 (C-2′), 128.80 (C-3′′, 5′′), 128.21 (C-4′), 128.18 (C-2′′, 6′′), 127.74 (C-3′), 124.00 (C-4a), 123.24 (C-6), 117.6 (C-8), 69.62 (OCH_2_); MS (ESI): m/z (M)^+^ = 667; Anal. Calc. For C_40_H_26_O_3_S; C, 72.07, H, 3.90, S, 9.61; found C, 72.13, H, 3.91, S, 9.63%.

#### A.5. Synthesis of 3,3′-(Biphenyl-4,4′-Diylbis(Methylene))Bis(Oxy)Bis(2-(Furan-2-yl)-4*H*-Chromen-4-One **2e**

The bischromenone **2e **was synthesized from the reaction of compound **1c** (2.00 g, 0.0082 mole) with 4,4′-bischloromethyl-diphenyl (1.02 g, 0.0041 mole) under similar condition as described above for **2a**.

Colorless solid; yield 25%; m.p.: 248–250°C; IR (KBr) cm^−1^: 1640 (C=O), 1605 (C=C); ^1^H-NMR (400 MHz, DMSO-d_6_): *δ* 8.20 (2H, dd, *J*_p,o_ = 1.5, 8.1 Hz, H-5), 7.81 (2H, d, *J*_3′,4′_ = 1.2 Hz, H-3′), 7.75 (2H, ddd, *J*_p,m,o_ = 1.5, 2.4, 7.8 Hz, H-7), 7.62 (10H, m, H-8, 2′′, 6′′, 3′′, 5′′), 7.45 (2H, t, *J*_o_ = 8.0 Hz, H-6), 7.34 (2H, d, *J*_5′,4′_ = 3.8 Hz, H-5′), 6.66 (1H, dd, *J*_5′,4′_ = 3.6 Hz, H-4′), 5.32 (4H, s, 3-OCH_2_);^ 13^C-NMR (DMSO-d_6_): *δ* 176.01 (C-4), 157.16 (C-8a), 141.92 (C-3), 140.16 (C-1′′), 138.91 (C-2), 135.23 (C-4′′), 135.11 (C-7), 130.59 (C-5), 130.49 (C-5′), 129.12 (C-2′), 128.81 (C-3′′, 5′′), 128.23 (C-4′), 128.10 (C-2′′, 6′′), 127.72 (C-3′), 124.02 (C-4a), 123.12 (C-6), 117.43 (C-8), 69.63 (OCH_2_); MS (ESI): m/z (M)^+^ = 635; Anal. Calc. For C_40_H_26_O_8_: C, 75.70, H, 4.10; found C, 75.47, H, 4.08%.

#### A.6. Synthesis of (4aS,5R)-5-(4′-((4aR,5S)-7-Oxo-2,4a,5,7-Tetrahydroisochromeno [4,3-b]Chromen-5-yl)Biphenyl-4-yl)-4a,5-Dihydroisochromeno [4,3-b]Chromen-7(2H)-One **3a**

A deoxygenated solution of bischromone **2a** (100 mg, 0.00015 mole) was carried out in dry IPA and dry THF with light from a 125 Hg arc lamp under nitrogen atmosphere in a pyrex vessel for 5 hours. The progress of reaction was monitored by TLC. The solvent was distilled out under reduced pressure, and the resulting photolysate thus obtained was subjected to extensive column chromatography (100–200 mesh), packed in petroleum ether-benzene (1 : 1). Elution of column with benzene-EtOAc (3 : 1) furnished starting compound** 2a **(14%, IR, co-tlc and mmp) and two new compounds **3a** and **4a** which were further recrystallized using MeOH.

Pale yellow solid; yield 22%; m.p.: 222–224°C; IR(KBr) cm^−1^: 1648 (C=O); ^1^H-NMR (400 MHz, DMSO-d_6_): *δ* 8.30 (2H, dd, *J*_p,o_ = 1.6, 8.2 Hz, H-7), 7.67 (2H, m, H-9), 7.63 (4H, dd, *J*_p,o_ = 1.0, 8.5 Hz, H-3′′, 5′′), 7.60 (2H, m, H-10), 7.58 (4H, dd, *J*_p,o_ = 1.0, 8.6 Hz, H-2′′, 6′′), 7.55 (2H, d, *J*_o_ = 8.1 Hz, H-8), 6.78 (2H, brs, H-1), 5.85 (2H, dd, *J*_3,2a_ = 1.2, *J*_3,4_ = 10.0 Hz, H-3), 5.30 (1H, dd, *J*_4,4a_ = 2.0, *J*_4,3_ = 10.3 Hz, H-4), 5.01 (2H, d,  *J*_5,4a_ = 11.0 Hz, H-5), 3.40 (2H, dd, *J*_4a,4_ = 2.0, *J*_4a,5_ = 11.0 Hz, H-4a), 2.95 (4H, m, H-2a); 13C-NMR (DMSO-d_6_): *δ* 177.82 (C-6), 155.52 (C-10a), 142.80 (C-6a), 141.50 (C-11b), 138.70 (C-4′′), 136.43 (C-1′′), 134.82 (C-9), 129.82 (C-4), 128.10 (C-3′′, 5′′), 127.62 (C-2′′, 6′′), 126.53 (C-1), 125.78 (C-7), 126.02 (C-3), 122.80 (C-8), 122.72 (C-11a), 117.02 (C-10), 82.5 (C-5), 38.02 (C-4a), 32.00 (C-2a); MS (ESI): m/z (M)^+^ = 655; Anal. Calc. For C_44_H_30_O_6_: C, 80.73; H, 4.58%; C, 80.97; H, 4.57%.

#### A.7. 5,5′-(Biphenyl-4,4′-Diyl)Diisochromeno [4,3-b]Chromen-7(5H)-One **4a**

Brown solid; Yield 24%; m.p.: 218–220°C; IR(KBr)cm^−1^: 1652 (C=O), ^1^H-NMR (400 MHz, DMSO-d_6_): *δ* 8.30 (2H, dd, *J*_p,o_ = 1.6, 8.0 Hz, H-7), 7.98 (2H, dd, *J*_m,o_ = 2.4, 8.9 Hz, H-1), 7.64 (2H, m, H-9), 7.62 (4H, dd, *J*_p,o_ = 1.0, 8.5 Hz, H-3′′, 5′′), 7.59 (2H, m, H-10), 7.57 (4H, dd, *J*_p,o_ = 1.0, 8.4 Hz, H-2′′, 6′′), 7.58 (2H, d, *J*_o_ = 8.0 Hz, H-8), 7.48 (4H, m, H-2, 3), 7.10 (2H, dd, *J*_m,o_ = 2.4, 8.5 Hz, H-4), 5.85 (2H, s, H-5); ^13^C-NMR (DMSO-d_6_): *δ* 180.02 (C-7), 156.62 (C-12a), 156.21 (C-11a), 139.00 (C-4′′), 136.04 (C-1′′), 134.82 (C-10), 128.32 (C-3′′, 5′′), 128.23 (C-1), 128.12 (C-3), 127.93 (C-8), 127.6 (C-2′′, 6′′), 127.19 (C-4a), 124.40 (C-4), 123.02 (C-2), 122.98 (C-9), 121.67 (C-6a), 113.68 (C-11), 72.93 (C-5); MS (ESI): m/z (M)^+^ = 648; Anal Calc. for C_44_H_26_O_6_: C, 81.23; H, 4.00; found C, 81.26; H, 4.16%.

#### A.8. Synthesis of (4aS,5R)-3-Methyl-5-(4′-((4aR,5S)-1-Methyl-7-Oxo-2,4a,5,7-Tetrahydroisochromeno [4,3-b]Chromen-5-yl)biphenyl-4-yl)-**4a**,Dihydroisochromeno-[4,3-b]Chromen-7(2H)-One **3b**

The photoirradiation of **2b **was carried out under similar condition as described above for **2a**. The resulting reaction mixture upon chromatographic workup yielded two new photoproducts **3b** and **4b**.

Light yellow solid; yield 32%; m.p.: 168-170°C; IR(KBr)cm^−1^: 1638 (C=O); ^1^H-NMR (400 MHz, DMSO-d_6_): *δ* 8.28 (2H, dd, *J*_p,o_ = 1.4, 8.1 Hz, H-7), 7.65 (2H, ddd, *J*_p,m,o_ = 1.0, 2.6, 8.5 Hz, H-9), 7.57 (2H, d, *J*_o_ = 8.0 Hz, H-8), 7.54 (2H, m, H-10) 7.53 (8H, m, H-2′′, 3′′, 5′′, 6′′), 6.81 (2H, brs, H-1), 5.25 (1H, dd, *J*_allyl,4,4a_ = 1.0, 1.9 Hz, H-4), 5.05 (2H, d, *J*_5,4a_ = 11.0 Hz, H-5), 3.45 (2H, dd, *J*_4,4a_ = 1.9, *J*_4a,5_ = 11.0 Hz, H-4a), 2.92 (4H, m, H-2a), 2.42 (3H, s, 4′-CH_3_); ^13^C-NMR (DMSO-d_6_): *δ* 177.20 (C-6), 157.20 (C-11), 143.50 (C-11b), 139.70 (C-4′′), 139.52 (C-1′′), 135.20 (C-9), 129.90 (C-4), 128.07 (C-3′′, 5′′), 127.62 (C-2′′, 6′′), 126.53 (C-1), 125.78 (C-7), 125.10 (C-3), 123.40 (C-8), 122.68 (C-11a), 118.72 (C-10), 81.5 (C-5), 39.02 (C-4a), 20.98 (3-CH_3_); MS (ESI): m/z (M)^+^ = 683; Anal Calc. For C, 80.93; H, 4.98; found C, 80.89; H, 4.96%.

#### A.9. 5,5′-(Biphenyl-4,4′-Diyl)Bis(3-Methylisochromeno [4,3-b]Chromen-7(5H)-One) **4b**

Light brown solid; yield 38%; m.p.: 179–181°C; IR(KBr)cm^−1^: 1638 (C=O); ^1^H-NMR (400 MHz, DMSO-d_6_): *δ* 8.26 (2H, dd, *J*_p,o_ = 1.3, 8.2 Hz, H-7), 7.91 (2H, d, *J*_o_ = 8.9 Hz, H-1), 7.63 (2H, ddd, *J*_p,m,o_ = 1.0, 2.5, 8.4 Hz, H-9), 7.55 (2H, d, *J*_*o*_ = 8.0 Hz, H-8),7.50 (2H, m, H-10), 7.48 (8 H, m, H-2′′, 3′′, 5′′, 6′′), 7.08 (4H, m, H-2, 4), 5.82 (2H, s, H-5), 2.43 (3H, s, 4′-CH_3_); ^13^C-NMR (DMSO-d_6_): *δ* 180.62 (C-7), 156.68 (C-12a), 156.24 (C-11a), 139.04 (C-4′′), 136.01 (C-1′′), 134.78 (C-10), 128.62 (C-3′′, 5′′), 128.23 (C-1), 128.01 (C-3), 127.98 (C-8), 127.62 (C-2′′, 6′′), 127.01 (C-4a), 124.42 (C-4), 123.08 (C-2), 122.95 (C-9), 121.58 (C-6a), 114.52 (C-11), 72.73 (C-5), 20.98 (3-CH_3_); MS (ESI): m/z (M)^+^ = 679; Anal Calc. for C_46_H_30_O_6_: C, 81.41; H, 4.42; found C, 81.39; H, 4.43%.

#### A.10. Synthesis of 5,5′-(Biphenyl-4,4′-Diyl)Bis(3-Methoxyisochromeno [4,3-b]Chromen-7(5H)-One **4c**

The deoxygenated THF solution of **2c** was photolysed under similar conditions as employed for **2a** and the chromatographic separation of photolysate furnished starting compound **2a **and compound **4c.**

Yellow solid; yield 30%; m.p.: 240-242°C; IR(KBr)cm^−1^: 1642 (C=O); ^1^H-NMR (400 MHz, DMSO-d_6_): *δ* 8.22 (2H, dd, *J*_p,o_ = 1.4, 7.8 Hz, H-7), 7.86 (2H, d, *J*_o_ = 8.7 Hz, H-1), 7.72 (2H, ddd, *J*_p,m,o_ = 1.0, 2.1, 8.3 Hz, H-9), 7.59 (2H, d, *J*_o_ = 8.2 Hz, H-8), 7.52 (8H, m, H-2′′, 3′′, 5′′, 6′′), 7.49 (2H, m, H-10), 6.95 (2H, m, H-2, 4), 5.78 (2H, s, H-5), 3.84 (6H, s, 4′-OC***H***_3_); 13C-NMR (DMSO-d_6_): *δ* 182.02 (C-6), 156.04 (C-11, 11a), 139.04 (C-4′′), 136.07 (C-1′′), 128.4 (C-3′′, 5′′), 128.13 (C-1), 127.82 (C-2), 127.52 (C-2′′, 4′′), 125.80 (C-3), 124.32 (C-4), 123.52 (C-2), 114.58 (C-11), 71.74 (C-5); MS (ESI): m/z (M)^+^ = 711; Anal Calc. for C_46_H_30_O_8_: C, 77.75; H, 4.22; found C, 77.98; H, 4.20%.

#### A.11. Synthesis of **5a**

The photolysis of **2d** under similar conditions as used for **2a** provided starting bischromone **2a** and two new products **5a** and **7**.

Light yellow solid; yield 32%; m.p.: 218-220°C; IR(KBr)cm^−1^: 1658 (C=O); ^1^H-NMR (400 MHz, DMSO-d_6_): *δ* 8.20 (2H, dd, *J*_p,o_ = 1.6, 8.0 Hz, H-7), 7.70 (2H, ddd, *J*_p,m,o_ = 1.0, 2.3, 7.9 Hz, H-9), 7.62 (4H, d, *J*_o_ = 8.0 Hz, H-3′′, 5′′), 7.55 (6H, m, H-10, 2′′, 6′′), 7.52 (2H, d, *J*_o_ = 8.1 Hz, H-8), 6.40 (2H, dd, *J*_2,3a_ = 1.0, *J*_2,3_ = 5.8 Hz, H-2), 5.45 (2H, d, *J*_4,3a_ = 10.0 Hz, H-4), 5.26 (2H, dd, *J*_3,3a_ = 3.5, *J*_3,2_ = 5.8 Hz, H-3), 5.10 (2H, d, *J*_11b,3a_ = 8.5 Hz, H-11b), 3.70 (2H, ddd, *J*_3,3a_ = 3.5, *J*_3a,11b_ = 8.5, *J*_3a,4_ = 10.0 Hz, H-3a); ^13^C-NMR (DMSO-d_6_): *δ* 178.20 (C-6), 156.92 (C-10a), 138.08 (C-4′′), 137.01 (C-1′′), 134.92 (C-9), 130.61 (C-11a), 129.62 (C-7), 128.52 (C-3′′, 5′′), 128.00 (C-2′′, 6′′), 127.7 (C-2), 124.61 (C-3), 123.42 (C-8), 122.80 (C-6a), 116.08 (C-10), 82.08 (C-4), 47.18 (C-3a), 33.52 (C-11b); MS (ESI): m/z (M)^+^ = 667; Anal Calc. for C_40_H_26_O_6_S_2_: C, 72.07; H, 3.90, S, 9.61; found C, 72.09; H, 3.87; S, 9.59%.

#### A.12. Synthesis of 7

Pale yellow solid; yield 21%; m.p.: 197–199°C; IR(KBr)cm^−1^: 1650 (C=O); ^1^H-NMR (400 MHz, DMSO-d_6_): *δ* 8.18 (2H, dd, *J*_p,o_ = 1.5, 8.1 Hz, H-7), 7.72 (2H, ddd, *J*_p.m,o_ = 0.9, 2.1, 8.2 Hz, H-9), 7.62 (2H, d,  *J*_2,3_ = 6.0 Hz, H-2), 7.59 (4H, d,  *J*_o_ = 8.2 Hz, H-3′′, 5′′), 7.54 (6H, m, H-10, 2′′, 6′′), 7.48 (2H, d, *J*_o_ = 8.3 Hz, H-8), 6.92 (2H, d, *J*_3,2_ = 6.0 Hz, H-3), 5.72 (2H, s, H-4); ^13^C-NMR (DMSO-d_6_): *δ* 178.92 (C-6), 155.58 (C-10), 141.18 (C-3), 140.92 (C-4), 139.72 (C-4′′), 137.41 (C-1′′), 135.02 (C-9), 134.82 (C-2), 132.18 (C-11b), 131.12 (C-11a), 128.12 (C-3′′, 5′′), 127.42 (C-2′′, 6′′), 124.82 (C-7), 123.08 (C-3), 122.82 (C-8), 115.98 (C-10), 73.58 (C-4); MS (ESI): m/z (M)^+^ = 663; Anal Calc. for C_40_H_22_O_6_S: C, 72.50; H, 3.32; S, 9.66; found C, 72.48; H, 3.30; S, 9.64%.

#### A.13. Synthesis of **6a**

The bischromone **2e** provided starting compound **2e** and one new photoproduct **6a** in THF under similar condition as used earlier for **2a**.

Light yellow solid; yield 28%; m.p.: 251–252°C; IR(KBr)cm^−1^: 1649 (C=O); ^1^H-NMR (400 MHz, DMSO-d_6_): *δ* 8.20 (2H, dd, *J*_p,o_ = 1.5, 8.1 Hz, H-7), 7.74 (2H, ddd, *J*_p,m,o_ = 1.4, 2.3, 7.9 Hz, H-9), 7.58 (10 H, m, H-10, 2′′, 3′′, 5′′, 6′′), 7.48 (2H, d,  *J*_*o*_ = 8.0 Hz, H-8), 6.43 (2H, brs, H-2), 5.48 (2H, d, *J*_4,3a_ = 11.0 Hz, H-4), 5.20 (2H, d,  *J*_3,3a_ = 1.0, *J*_3,2_ = 2.7 Hz, H-3), 5.06 (2H, d, *J*_11b,3a_ = 8.2 Hz, H-11b), 3.67 (2H, ddd, *J*_3a,3_ = 1.0, *J*_3a,11b_ = 8.8, *J*_3a,4_ = 11.0 Hz, H-3a); ^13^C-NMR (DMSO-d_6_): *δ* 177.68 (C-6), 156.88 (C-10a), 136.92 (C-4′′), 136.01 (C-1′′), 134.82 (C-9), 130.52 (C-11a), 129.58 (C-7), 128.48 (C-3′′, 5′′), 128.11 (C-2′′, 6′′), 127.68 (C-2), 124.58 (C-3), 123.38 (C-8), 122.72 (C-6a), 116.12 (C-10), 82.10 (C-4), 47.12 (C-3a), 33.48 (C-11b); MS (ESI): m/z (M)^+^ = 635; Anal Calc. for C_40_H_26_O_8_: C, 75.70; H, 4.10; found C, 75.92; H, 4.11%.
